# Construct Validity and Clinical Utility of World Health Organization Disability Assessment Schedule 2.0 in Older Patients Discharged From Emergency Departments

**DOI:** 10.3389/fresc.2021.710137

**Published:** 2021-08-17

**Authors:** Louise Moeldrup Nielsen, Lisa Gregersen Oestergaard, Hans Kirkegaard, Thomas Maribo

**Affiliations:** ^1^Department of Occupational Therapy, Research Centre for Health and Welfare Technology, VIA University College, Aarhus, Denmark; ^2^Department of Physiotherapy and Occupational Therapy, Aarhus University Hospital, Aarhus, Denmark; ^3^Department of Public Health, Aarhus University, Aarhus, Denmark; ^4^DEFACTUM Central Denmark Region, Aarhus, Denmark; ^5^Research Centre for Emergency Medicine, Emergency Department, Department of Clinical Medicine, Aarhus University Hospital, Aarhus University, Aarhus, Denmark

**Keywords:** WHODAS 2.0, older patients, functioning, ICF, rehabilitation

## Abstract

**Introduction:** The World Health Organization Disability Assessment Schedule 2.0 (WHODAS 2.0) is designed to measure functioning and disability in six domains. It is included in the International Classification of Diseases 11th revision (ICD-11). The objective of the study was to examine the construct validity of WHODAS 2.0 and describe its clinical utility for the assessment of functioning and disability among older patients discharged from emergency departments (EDs).

**Material and Methods:** This cross-sectional study is based on data from 129 older patients. Patients completed the 36-item version of WHODAS 2.0 together with the Barthel-20, the Assessment of Motor and Process Skills (AMPS), Timed Up and Go (TUG), and the 30-Second Chair Stand Test (30 s-CST). Construct validity was examined through hypothesis testing by correlating the WHODAS with the other instruments and specifically the mobility domain in WHODAS 2.0 with the TUG and 30 s-CST tests. The clinical utility of WHODAS 2.0 was explored through floor/ceiling effect and missing item responses.

**Results:** WHODAS 2.0 correlated fair with Barthel-20 (*r* = −0.49), AMPS process skills (*r* = −0.26) and TUG (*r*=0.30) and correlated moderate with AMPS motor skills (*r* = −0.58) and 30s-CST (*r* = −0.52). The WHODAS 2.0 mobility domain correlated fair with TUG (*r* = 0.33) and moderate with 30s-CST (*r* = −0.60). Four domains demonstrated floor effect: D1 “Cognition,” D3 “Self-care,” D4 “Getting along,” and D5 “Household.” Ceiling effect was not identified. The highest proportion of missing item responses were present for Item 3.4 (Staying by yourself for a few days), Item 4.4 (Making new friends), and Item 4.5 (Sexual activities).

**Conclusion:** WHODAS 2.0 had fair-to-moderate correlations with Barthel-20, AMPS, TUG, and 30s-CST and provides additional aspects of disability compared with commonly used instruments. However, the clinical utility of WHODAS 2.0 applied to older patients discharged from EDs poses some challenges due to floor effect and missing item responses. Accordingly, patient and health professional perspectives need further investigation.

## Introduction

Identifying the level of disability among older patients hospitalized with a medical diagnosis is an essential component of their treatment, as it is used to drive the discharge planning process and possible referral to rehabilitation (1). Discharge planning often requires a multidisciplinary approach and involves a tailored plan for the patient to facilitate prompt and efficient discharge. Accordingly, instruments measuring different aspects of disability are used in a clinical context (2–4), including the Barthel Index, the Functional Independence Measure (FIM), the KATZ ADL Index, the 30-Second Chair Stand Test (30s-CST), and the Timed Up and Go (TUG) (2–4).

The World Health Organization Disability Assessment Schedule 2.0 (WHODAS 2.0) (5) is based on the International Classification of Functioning (ICF) framework (6). WHODAS 2.0 is a generic patient-reported instrument that measures functioning and disability. The use of WHODAS 2.0 is recommended as suitable for describing and quantifying the level of disability associated with a health condition and is included in the new International Classification of Disease 11th revision (ICD-11) (7, 8). WHODAS 2.0 is a generic, multi-dimensional questionnaire that rates functioning from the respondent's subjective perspective. It enables comparison across different groups and settings for six different functional domains that reflect a hierarchy of disability, which is especially useful for clinical purposes and in research (5). There are different modes and versions of WHODAS 2.0, including 12- and 36-item versions, with the instrument having been translated into more than 40 languages (5, 8–11).

A number of studies have been conducted that examine the reliability and validity of the WHODAS 2.0 among different populations (5, 9, 12–14). In a sample of 1,190 patients with chronic diseases, the 36-item interviewer-based version demonstrated high reliability and a good ability to discriminate and detect change over time (12). Additionally, the 36-item version were found to have high reliability and validity in a sample of 1,000 elderly people (60–70 year) in Poland (10). In a systematic review of 810 studies, the authors concluded that WHODAS 2.0 offers a valid, reliable, self-report measure of disability for a variety of populations and settings (8).

Although the psychometric properties of WHODAS 2.0 seem solid, the validity and clinical utility of WHODAS 2.0 among older patients with a medical diagnosis in an emergency department (ED) setting has not yet been explored. Accordingly, the objective of this study was to examine the construct validity of WHODAS 2.0 and to describe its clinical utility for assessing disability and functioning among older patients discharged from EDs.

## Materials and Methods

The present study adhered to the STROBE guidelines for standard of reporting (15).

### Study Design

This cross-sectional study is based on baseline data from a previous non-randomized controlled trial including older patients (16). The objective of the trial was to examine the effectiveness of an intervention aimed at reducing the risk of readmission among older patients discharged from the ED. The intervention consisted of an assessment of patients' limitations in performing daily activities, referral to further rehabilitation in primary care, and a follow-up visit at home the day after discharge (16).

### Setting

The study took place at an emergency department at a 1.150-bed University hospital in Denmark. Patients were included from March to December 2014.

### Participants

Inclusion criteria for participants were as follows: people aged ≥65 years admitted with an acute medical diagnosis to the ED on weekdays only. Those who were admitted from a nursing home, transferred to other hospital departments, unable to communicate, and declared terminally ill were excluded. In this study, we use baseline data from patients in the intervention group. All participants included in the study gave written consent for their enrolment. The study was approved by the Danish Data Protection Agency (J.nr. 2012-41-0763) and by the Danish Health Authority (3-3013-608/1/).

### Data Sources and Measurement

***The World Health Organization Disability Assessment Schedule 2.0 (WHODAS 2.0)*** The 36-item interviewer-based version was used except for the four items regarding employment, as most of the patients were retired. WHODAS 2.0 is designed to evaluate functioning in six domains: D1 “Cognition,” D2 “Mobility,” D3 “Self-care,” D4 “Getting along,” D5 “Life activities” (items related to work are not included), and D6 “Participation.”

Participants were asked to indicate their experienced level of difficulty over the preceding 30 days using a 5-point rating scale by taking into account the way in which they normally perform a given activity and including the use of whatever support and/or help from either a person or the use of aids. A standardized algorithm that weights the items and the level of severity (17) was used to determine the score, ranging from 0 to 100 (with high scores indicating greater disability). Missing data were handled in accordance with the WHODAS 2.0 manual: the mean scores across all items within the domain were assigned to the missing item response (17).

***Barthel-20*** is one of the most commonly used measures of functioning in older patients (18). The instrument measures a person's level of independence in performing daily activities. The scale is ordinal and comprises ten basic activities (grooming, bathing, feeding, getting on and off the toilet, ascending and descending stairs, getting dressed, bladder incontinence, bowel incontinence, walking, and transferring). Barthel has been evaluated in different settings with older patients with acceptable psychometric properties (18, 19). A widely adopted modification, the Barthel-20 uses a score range from 0 (high dependence on assistance) to 20 (independent of assistance). In this study, the Barthel-20 was used as a self-report instrument conducted through interviews (20, 21). Participants with missing data were excluded from the analyses.

***Assessment of Motor and Process Skills*** is a standardized, observation-based, occupational therapy instrument that measures the quality of a person's performance of daily activities in a natural and task-relevant environment. Quality is determined by the person's effort, efficiency, safety, and independence in performing two different tasks. The AMPS consists of two scales, one measuring motor skills and one measuring process skills. The quality of each skill is scored on a 4-point ordinal scale and then converted into an overall mean score for motor and process abilities, using the AMPS software (22, 23). AMPS has been evaluated in different settings with older patients with acceptable psychometric properties (23, 24). As AMPS are observation based there are no missing data.

***Timed Up and Go*** was originally described as a mobility test for frail older persons. TUG is widely used, it is simply to apply in a clinical context and it is recommended to use in Geriatric Emergency Medicine Guidelines (25, 26). It reflects a person's ability to get up from an armchair, walk three meters, return, and sit down. Participants were asked to walk as fast and safely as possible while wearing regular footwear. If needed, the participants were allowed to use their customary walking aid. The faster a person can move, the better. A score of <20 s reflects independence in basic transfer (27). TUG has been evaluated in different settings with older patients with acceptable psychometric properties (28). No missing data exist in the TUG.

***Thirty-Second Chair Stand Test*** is a physical performance instrument that assesses lower body strength as an important proxy for mobility. The simplicity of the test makes it easy to use, requiring <5 min. The test was administered using a chair with no arm rest. When given the signal to “go,” the participant rose to a full standing position and was then instructed to complete as many full stands as possible within 30 s. A low score (<8), indicates disability (4). 30 s-CST has been evaluated in different settings with older patients with acceptable psychometric properties (4, 29) and the Danish Health Authorities recommend the instrument to be used in clinical contexts (30). No missing data exist in 30-CST.

#### Patient Characteristics

Demographic and clinical variables such as age, gender, marital status, days of admission, and comorbidity measured with the Charlson Comorbidity Index (CCI) (31) were extracted from the Danish National Patient Registry.

### Procedures for Measurement

Interviews were conducted using the WHODAS 2.0 and Barthel-20 by occupational therapists with experience in the acute care area and who had been trained to administer these specific instruments. After interviewing the participant, the occupational therapist performed the AMPS (16). Next, a physiotherapist performed the 30 s-CST and TUG (16).

The occupational therapists and physiotherapists participated in a 2-week training period to ensure correct implementation of both the interview-based and performance-based tests prior to the inclusion of participants. The training included review of written instructions, repeated practice in using the tests, and supervision (16).

### Analytical Strategy

The terminology and concepts proposed by the Consensus-based Standards for the Selection of Health Measurement Instruments (COSMIN) were applied (32). Construct validity based on hypothesis testing is defined as “*the degree to which the scores of a measurement instrument are consistent with hypotheses, regard relationships with scores of other instruments*” (32). A priori hypotheses were tested based on the assumption that instruments that represent the same construct would be moderate correlated, while instruments that measure different aspects of the construct would be fair correlated.

To identify similarities and differences between the constructs of the instruments, we provided an overview of how the instruments were linked to the ICF (See Table 1) (17, 33–36).

**Table 1:** Links between first-level ICF categories and measurement instruments.

**ICF: Activities and participation**	**WHODAS 2.0 (17)**	**Barthel-20 (36)**	**AMPS (35)**	**TUG (34)**	**30 s-CST (34)**
d1 Learning and applying knowledge			X		
d2 General tasks and demands	X		X		
d3 Communication	X				
d4 Mobility	X	X	X	X	X
d5 Self-care	X	X	X		
d6 Domestic life	X		X		
d7 Interpersonal interaction and relationships	X				
d8 major life areas	(X)				
d9 Community, social and civic life	X				

Based on the linking to ICF, we expect WHODAS 2.0 to describe the construct of functioning in broader terms than the other instruments, and hypothesize a fair correlation (*r* = 0.25–0.49) between WHODAS 2.0 and the following five instruments: Barthel-20, AMPS motor scale, AMPS process scale, TUG, and 30 s-CST. We expect the WHODAS 2.0 domain D2 “mobility” to be more closely correlated to TUG and 30 s-CST as their constructs are related to mobility and thus, hypothesize a moderate correlation (*r* = 0.50–0.74).

The clinical utility of WHODAS 2.0 was explored by analyzing floor and ceiling effects and subgroup analysis of missing item responses. Missing responses in WHODAS 2.0 were analyzed before replacing the missing value with mean score across the other items in the domain.

### Statistical Methods

Descriptive statistics were used to present the characteristics of the study population. Frequencies and proportions were reported for categorical variables. For the continuous variables, the median and interquartile range (IQR) were used for skewed data, while the mean and standard deviation (SD) were used for normally distributed data.

Construct validity was estimated using either Pearson's or Spearman's correlation coefficient (as appropriate) with 95% confidence intervals (CI). Interpretation of the correlation coefficients was based on the following: fair (*r* = 0.25–0.49), moderate (*r* = 0.50–0.74), and excellent (*r* ≥ 0.75) (37).

Floor and ceiling effects were examined through descriptive statistics and subgroup analyses. Such effects occur if more than 15% of patients achieve either the lowest or highest possible score (32). Subgroup analyses were conducted to explore whether participants with more than 15% missing item responses were different from the rest of the group. All tests were two-tailed, assuming a 5% significance level. Analyses were performed using STATA 15.

## Results

### Participants

In total, 179 patients aged 65 years or more were invited to participate, of whom 144 (80%) agreed to take part [see flowchart in (16)]. Due to more than two missing item responses in some of the WHODAS domains, 15 participants were excluded, resulting in a study sample of 129 participants for this study. There were no significant differences between participants and excluded patients in relation to age, gender, comorbidity score, AMPS, or TUG. Significant differences were found for Barthel-20 and 30 s-CST (see Supplementary Material). Descriptive statistics for the study sample are presented in Table 2.

**Table 2:** Characteristics of the participants (*n* = 129).

Characteristics	
Mean age, years (SD)	80.4 (7.8)
Female, *n* (%)	68 (53%)
**Marital status**, ***n*** **(%)**	
Widowed	43 (33%)
Divorced	28 (22%)
Married	52 (40%)
Single	6 (5%)
**Comorbidity**, ***n*** **(%)**	
Low: score 0–1	66 (51%)
Moderate: score 2–3	42 (33%)
High: score >4	21 (16%)
Days of admission, mean (SD)	1.123 (0.69)
WHODAS 2.0 sum score, mean (SD)^*^	25.3 (17.0)
Barthel-20, median (IQR)^#^^a^	19 (17–20)
AMPS motor, mean (SD)^#^^b^	1.08 (0.80)
AMPS process, mean (SD)^#^^b^	0.90 (0.85)
TUG score, mean (SD)^*^^c^	15.2 (10.7)
30 s-CST, mean (SD)^#^^d^	6.6 (4.8)

* *High score indicates severe disability*.

# *Low score indicates severe disability*.

a *Barthel-20 n = 125*.

b *Assessment of motor and process skills n = 83*.

c *Timed up and go n = 110*.

d *30 s-Chair Stand Test n = 116*.

### Main Results

Table 3 presents the correlations between the sum scores for the WHODAS 2.0 and the other instruments. Fair correlations were found with the Barthel-20, AMPS process skills and TUG, while moderate correlations were found with the AMPS motor skills and 30 s-CST. For the WHODAS 2.0 mobility domain, a fair correlation was found with TUG, while the correlation with 30 s-CST was moderate.

**Table 3:** Correlation with 95% confidence intervals between WHODAS 2.0 and other measures of functioning.

	**WHODAS 2.0**	**D2. Mobility**
Barthel-20^#^^a^	−0.49^*^ (−0.63; −0.34)	
AMPS motor^¤^*^b^*	−0.58^*^ (−0.72; −0.43)	
AMPS process^¤^*^b^*	−0.26^*^ (−0.48; −0.04)	
Timed Up and Go^¤^*^c^*	0.30^*^ (0.11;0.50)	0.33^*^ (0.16;0.49)
30 s. Chair Stand Test^¤^*^d^*	−0.52^*^ (−0.65; −0.40)	−0.60^*^ (−0.71; −0.49)

# *Spearman correlation*.

¤ *Pearson's correlation*.

a *Barthel-20 n = 125*.

b *Assessment of motor and process skills n = 83*.

c *Timed up and go n = 110*.

d *30 s-Chair Stand Test n = 116*.

* *p < 0.05*.

As more than 15% of participants exhibited either floor or ceiling effect in the Barthel-20 and 30 s-CST (Table 4), secondary analysis was performed excluding participants with a score of 20 for the Barthel-20 and a score of zero in the 30 s-CST. This did not change the overall result, as the correlation between the WHODAS 2.0 and the Barthel-20 remained fair [*r* = −0.26 (95%CI −0.51; 0.001)] and the 30 s-CST moderate [*r* = −0.52 (95%CI −0.66; −0.38)].

**Table 4:** Distribution of the instruments score.

**Domain**	**Mean (SD)**	**Median (IQR)**	**Range**	**Missing, *n* (%)**	Floor^*^, *n* (%)	**Ceiling^#^, *n* (%)**
WHODAS 2.0*^a^*	25.3 (17.0)	22.8 (10.9–36.9)	0–69	–	1 (0.8%)	0
D1. Cognition	16.0 (16.6)	10.0 (5.0–25.0)	0–70	5 (4%)	27 (21%)	0
D2. Mobility	36.7 (28.5)	31.3 (12.5–62.5)	0–94	0 (0%)	15 (12%)	0
D3. Self-care	20.0 (23.2)	10.0 (0.0–30.0)	0–90	25 (19%)	47 (36%)	0
D4. Getting along	19.1 (21.5)	16.7 (0.0–33.3)	0–83	51 (40%)	47 (36%)	0
D5. Life-activities	33.8 (30.5)	30.0 (10.0–50.0)	0–100	3 (2%)	27 (21%)	7 (5%)
D6. Participation	27.2 (19.3)	25.0 (12.5–37.5)	0–83	15 (12%)	9 (7%)	0
Barthel-20*^b^*	18.3 (2.3)	19 (17–20)	8–20	–	0	55 (44%)
AMPS motor*^c^*	1.08 (0.80)	1.09 (0.6–1.6)	−1.7 to 2.6	–	–	–
AMPS process*^c^*	0.90 (0.85)	1.08 (0.6–1.4)	−3.8 to 2.6	–	–	–
Timed Up and Go*^c^*	15.2 (10.7)	11.1 (8.7–17.4)	5.2–60.2	–	–	–
30s. Chair Stand Test*^e^*	6.6 (4.8)	7 (2–10)	0–19	–	28 (24%)	–

a *WHODAS 2.0, n = 129*.

b *Barthel-20 n = 125*.

c *Assessment of motor and process skills n = 83*.

d *Timed up and go n = 110*.

e *30 s-Chair Stand Test n = 116*.

* *Percentages that scores lowest possible score*.

# *Percentages that scores highest possible score*.

Mean score of WHODAS 2.0 domains are presented in Table 4. Due to high SD, median and IQR are also presented. Missing item responses were present in all WHODAS 2.0 domains except for D2 “Mobility.” The highest proportion of missing responses were present in Item 3.4 (Staying by yourself for a few days) (18.6%), Item 4.4 (Making new friends) (17.9%), and Item 4.5 (Sexual activities) (31.8%) (see Supplementary Material).

Participants with missing responses had a significantly lower score in Barthel-20 than participants who provided a response (18 vs. 19, *p* < 0.05). For Item 3.4, participants with missing responses had significantly higher scores in AMPS motor (1.38 vs. 0.97, *p* < 0.05) and process skills (1.35 vs. 0.75, *p* < 0.05) than participants who responded. For Item 4.5, participants with missing responses were significantly older (83.2 vs. 79.2, *p* < 0.05) than participants who responded (see Supplementary Material).

Floor effect, indicating no disability, was identified in four WHODAS 2.0 domains: D1 “Cognition” (21%), D3 “Self-care” (36%), D4 “Getting along” (36%) and D5 “Life-activities” (21%), as shown in Table 3. A significant difference between participants with the lowest possible score (floor effect) and other participants was found in relation to the AMPS motor skills and 30 s-CST scores, where participants with a score of zero in the domains had higher scores in both AMPS motor skills and 30 s-CST.

In domain D3 “Self-care,” participants with a score of zero were significantly younger, had a higher Barthel-20 score, and a lower score in TUG than other participants. A significantly higher Barthel-20 score was also identified for participants with a score of zero in domain D4 “Getting along,” compared to other participants. In domain D5 “Life-activities,” differences were found between participants with a score of zero and other participants in relation to age, gender, Barthel-20, AMPS process skills and TUG (Supplementary Material). Ceiling effect was not found in any of the WHODAS 2.0 domains, meaning that none of the participants reported severe disability.

## Discussion

The current study is the first to examine the construct validity and clinical utility of the WHODAS 2.0 36-item version in a sample of older patients discharged from EDs. The results demonstrate fair-to-moderate correlations between WHODAS 2.0 sum scores and WHODAS 2.0 mobility domains and the Barthel-20, AMPS, TUG, and 30 s-CST instruments. Floor effect and missing item responses were present in four domains: D1 “Cognition,” D3 “Self-care,” D4 “Getting along,” and D5 “Life-activities” while missing item responses were identified in Items 3.4, 4.4, and 4.5.

We expected a priori that the correlation between WHODAS 2.0 sum score and the Barthel-20, AMPS, TUG, and 30 s-CST would be fair, while the correlation between the WHODAS 2.0 mobility domain and the other instruments measuring mobility would be moderate. However, the results revealed that due to varied CIs, all the correlations (except the correlation between WHODAS 2.0 and AMPS process skills) were either fair or moderate. Our hypotheses cannot therefore be verified. The highest correlations (moderate) with WHODAS 2.0 were found with the AMPS motor skills and 30 s-CST, while the highest correlation for the mobility domain was found with 30 s-CST. The fair-to-moderate correlations between the WHODAS 2.0 sum scores and the other well-established instruments may indicate similarity of constructs but also that the WHODAS 2.0 is measuring other aspects of functioning and disability. The linking of the instruments to ICF shows that the WHODAS 2.0 are covering a broader aspect of functioning than any of the other instruments (17). WHODAS 2.0 are covering elements from seven activity and participation domains (domain 2–7 + 9) and sparse elements from one domain (domain 8), while the other instruments covers fewer domains. The 30 s-CST and TUG only cover the domain mobility, and are thus seen as unidimensional instruments. In other studies that examine the construct validity of WHODAS 2.0, correlations have also been reported mainly as fair-to-moderate compared with other multidimensional instruments such as the Short Form 36 (14, 38). In those studies, with fair to moderate correlation, the authors conclude that their results provide evidence for the validity of WHODAS 2.0 (14, 38). However, it can be questioned whether a fair-to-moderate correlation should be considered an expression of validity or rather an expression of different instruments measuring related but different constructs (39). The trade off between using a multidimensional or unidimensional instrument of disability must be careful considered in a clinical context with high patient flow, but our results indicate that the use of a multidimensional instrument such as WHODAS 2.0 provides additional aspects of disability compared with commonly used instruments in this population.

We identified a mean sum score of 25.3 with wide SDs (SD 17.0) for the WHODAS 2.0. In other studies, similar mean scores were identified for patient samples with different diagnoses and disabilities. In one validity study, a mean sum score of 22.9 (SD 16.1) was found in a younger (but disabled) population (40). In another study from 2017, a mean sum score of 30.9 (SD 16.2) was reported in a sample of patients at a specialized somatic rehabilitation clinic (38), while another study (12) identified a mean score of 24.8 (SD 19.3) in a sample of 1,119 patients with chronic conditions. Whether a mean score of 25.3 is low or high depends on the population. To our knowledge, no normative score for an older population with the 36-item version of the instrument is available. However, in (40), the sample of disabled people was compared with a sample of people with no reported disabilities. The mean WHODAS 2.0 sum scores were found to be significantly different in the two groups (22.9 for the disabled group compared with 12.9 in the group not reporting disability).

We found floor effect in the following domains: D1 “Cognition,” D3 “Self-care,” D4 “Getting along,” and D5 “Life-activities.” Participants with a domain score of zero—indicating no disability—had a significantly higher score in the AMPS motor skills, 30 s-CST, and Barthel-20 (D3, D4, and D5) and a lower score in TUG (D3 and D5). In relation to age, participants with a score of zero in D3 and D5 were significantly younger than other participants. Floor effect has also been reported in other studies. In a study from 2014 (9), the authors reported floor effect in the D4 and D5 domains, while another study reported floor effect in the D3 and D2 domains (40). When floor effect occurs, it reduces the variability of the instrument and may therefore affect the validity. However, we found consistency between domains with floor effect and the scores of the other instruments indicating no disability. We found no ceiling effect, which is in contrast with other studies that have reported ceiling effect for the WHODAS 2.0 (12, 41). This means that none of the participants reported severe disability. Both floor and ceiling effects are important when it comes to the clinical utility of an instrument. An instrument with ceiling or floor effect hampers the possibility to detect change in disability over time

In Items 3.4, 4.4, and 4.5, more than 15% of the participants had missing responses.

For all three items, these participants had a significantly lower score for the Barthel-20 than participants who responded. This indicates that participants with lower functioning found in Barthel-20 were more likely to have missing responses. The highest proportion of missing responses was found for Item 4.5 (Sexual activities), with 32% of the sample having missing responses. This is in accordance with other studies (9, 12) that also report a high proportion of missing values for this item. A possible reason may be that for some, the issue of sexual activity is considered either a private matter or not relevant. In this sample, 40% were married, while 60% were either widowed, divorced, or single. Although this may have influenced participants' responses, our subgroup analysis revealed that there were no significant differences between responses and missing responses in relation to marital status (Supplementary Material). More than 15% missing responses in item 3.4 and 4.4 may be related to the relevance of the questions. Staying by yourself for a few days (Item 3.4) and Making new friends (Item 4.4) may not have been relevant for a part of this population in the last 30 days. The reasons for not responding to certain items for this population need further examination.

The relatively high proportion of missing responses in the three items indicates that completion of the WHODAS 2.0 36-item version may pose some challenges for this population, which may hamper the clinical utility. Instead, the 12-item version (not including these three items) may be easier to apply. A study from 2020 (13) reported no missing responses in the validation of the 12-item version with an older population. However, that study was an epidemiological survey and not conducted in a clinical context with patients discharged from hospital. The 36-item version is more comprehensive; accordingly, using the 12-item version may result in less information across the different domains, information that may otherwise be useful in a clinical context where patients' further rehabilitation needs to be planned prior to discharge. Whether the 12-item version might be more suitable than the 36-item version among the older population in a clinical context needs to be explored in future studies.

Another aspect that needs further examination among this population is the use of a timeframe of 30 days when answering the questions, which may be problematic. Older patients with an acute admission to an ED may have experienced a sudden, but unrecognized disability that could influence the accuracy of their self-reported functioning, leading to an underestimation of their disabilities (16). Whether the WHODAS 2.0 is able to detect sudden disabilities, an issue of importance in a clinical context, should be further explored. This is highly relevant, especially as the instrument is included in the ICD-11.

Although examining the clinical utility of WHODAS 2.0 in a population of older patients at the ED is new, its utility in other populations has been examined extensively in the recent years (42–44). The WHODAS 2.0 was found a useful measure of disability in a population with chronic pain (42) and for stroke survivors (43) were WHODAS 2.0 showed good reliability and validity. In addition, the WHODAS 2.0 has also been found useful for valid interpretations of disability in people with psychiatric health conditions (44).

## Strengths and Limitations of the Study

The construct validity of WHODAS 2.0 was measured in this study with hypotheses testing. Construct validation is often considered less powerful than criterion validation; however, when no gold standard is present, hypothesis testing can be used to examine whether the instrument measures what it is supposed to measure (32). The sample included in this study was above the number (*n* = 50) recommended as a minimum (45) for validity studies, although lower than some comparable studies (14, 38). The relatively small sample size affects the CIs and thereby the uncertainty of the results.

Clinical utility of the WHODAS 2.0 were examined through floor and ceiling effect and subgroup analysis exploring participants with more than 15% missing item response. For clinical use, it would however, have been relevant to examine the responsiveness of WHODAS 2.0. This was not possible due to the cross-sectional study design.

Another limitation of the study is the generalization of the results. The sample included only older patients discharged from EDs, which may hamper generalization. The limitation of generalization emphasizes the importance of continuing to study the value and psychometric properties of the WHODAS 2.0 in samples of patients treated in different settings and with different health conditions.

## Conclusion

In conclusion, WHODAS 2.0 demonstrated fair-to-moderate correlations with the Barthel-20, AMPS, TUG, and 30 s-CST instruments. The results indicate that WHODAS 2.0 provides a different aspect of functioning and disability than instruments commonly used with older patients. WHODAS 2.0 provides value in a clinical context, as it is distinguished from other instruments as being a measure that applies the ICF biopsychosocial approach. However, the clinical utility of the WHODAS 2.0, used with a population of older patients discharged from EDs, also poses some challenges due to floor effect in four of the domains and due to missing responses for three items. Together with its compatibility with the ICD-11, the WHODAS 2.0 is expected to become widely used in clinical contexts; however, its utility from patient and health professional perspectives need further investigation.

## Data Availability Statement

The raw data supporting the conclusions of this article will be made available by the authors, without undue reservation.

## Ethics Statement

The study was approved by the Danish Data Protection Agency (J.nr. 2012-41-0763) and by the Danish Health Authority (3-3013-608/1/). All participants included in the study gave written consent for their enrolment.

## Author Contributions

LN, LO, HK, and TM: study conception and design and critical revision. LN and TM: analysis and interpretation of data. LN: drafting of manuscript. All authors contributed to the article and approved the submitted version.

## Conflict of Interest

The authors declare that the research was conducted in the absence of any commercial or financial relationships that could be construed as a potential conflict of interest.

## Publisher's Note

All claims expressed in this article are solely those of the authors and do not necessarily represent those of their affiliated organizations, or those of the publisher, the editors and the reviewers. Any product that may be evaluated in this article, or claim that may be made by its manufacturer, is not guaranteed or endorsed by the publisher.
