# Supplementary material for: Construct Validity and Clinical Utility of World Health Organization Disability Assessment Schedule 2.0 in Older Patients Discharged From Emergency Departments
Source: Front Rehabil Sci. 2021 Aug 17;2:710137. doi: 10.3389/fresc.2021.710137 (PMC9397984; doi:10.3389/fresc.2021.710137)
Supplement: Supplementary file 1 [file Data_Sheet_1.docx]

**Supplementary material**

**Supplementary Table 1.** Overview of WHODAS 2.0 items and missing response (n=129)

| Items | Number of persons with Missing in the item |
| --- | --- |
| D1. Cognition |  |
| D 1.1 | 0 |
| D 1.2 | 1 |
| D 1.3 | 0 |
| D 1.4 | 3 |
| D 1.5 | 0 |
| D 1.6 | 1 |
| D2. Mobility |  |
| D 2.1 | 0 |
| D 2.2 | 0 |
| D 2.3 | 0 |
| D 2.4 | 0 |
| D 2.5 | 0 |
| D3. Self-care |  |
| D 3.1 | 0 |
| D 3.2 | 1 |
| D 3.3 | 0 |
| D 3.4 | 24 |
| D4. Getting along |  |
| D 4.1 | 2 |
| D 4.2 | 0 |
| D 4.3 | 0 |
| D 4.4 | 23 |
| D 4.5 | 41 |
| D5. Life-activities |  |
| D 5.1 | 0 |
| D 5.2 | 0 |
| D 5.3 | 2 |
| D 5.4 | 1 |
| D6. Participation |  |
| D 6.1 | 6 |
| D 6.2 | 1 |
| D 6.3 | 0 |
| D 6.4 | 3 |
| D 6.5 | 0 |
| D 6.6 | 0 |
| D 6.7 | 4 |
| D 6.8 | 3 |

**Supplementary Table 2**. Differences between study participants and excluded due to missing response.

|  | Study participants (n=129) | Excluded (n=15) | Difference |
| --- | --- | --- | --- |
| Mean age, years (SD) | 80.4 (7.8) | 82.8 (2.4) | p=0.28 |
| Female, n (%) | 68 (53%) | 11 (73%) | p=0.13 |
| Comorbidity, n (%)  Low: score 0-1  Moderate: score 2-3  High: score >4 | 66 (51%)  42 (33%)  21 (16%) | 9 (60%)  3 (20%)  3 (20%) | p=0.61 |
| Barthel-20, median (IQR)  AMPS motor, mean (SD)  AMPS process, mean (SD)  TUG score, mean (SD)  30s-CST, mean (SD) | 19 (17-20)  1.08 (0.80)  0.90 (0.85)  15.2 (10.7)  6.6 (4.8) | 17 (14-18)  0.68 (0.59)  1.10 (0.31)  16.4 (5.3)  2.8 (3.2) | p=0.002  p=0.08  p=0.40  p=0.71  p=0.02 |

**Supplementary Table 3.** Differences between participants with response compared to participants with missing response

|  | Item 3.4 | | Item 4.4 | | Item 4.5 | |
| --- | --- | --- | --- | --- | --- | --- |
|  | Response  n=105 | Missing  n=24 | Response n=106 | Missing n=23 | Response n=88 | Missing n=41 |
| Mean age, years (SD) | 80.2 (8.0) | 81.3 (6.7) | 80.1 (7.8) | 81.8 (7.3) | 79.2 (7.9)* | 83.2 (6.8)* |
| Female, n (%) | 54 (49.5%) | 16 (66.7%) | 54 (50.9%) | 14 (60.9%) | 42 (47.7%) | 26 (63.4) |
| Marital status, n (%) |  |  |  |  |  |  |
| Widowed | 29 (27.6%)* | 14 (58.3%)* | 36 34.0%) | 7 (30.4%) | 24 (27.3%) | 19 (46.3%) |
| Divorced | 24 (22.9%)* | 4 (16.7%)* | 20 (18.9%) | 8 (34.8%) | 20 (22.7%) | 8 (19.5%) |
| Married | 47 (44.8%)* | 5 (20.8%)* | 45 (42.4%) | 7 (30.4%) | 40 (45.6%) | 12 (29.3%) |
| Single | 5 (4.8%)* | 1 (4.2%)* | 5 (4.7%) | 1 (4.4%) | 4 (4.6%) | 2 (4.9%) |
| Comorbidity, n (%) |  |  |  |  |  |  |
| Low: score 0-1 | 51 (48.6%) | 15 (62.5%) | 54 (50.9%) | 12 (52.2%) | 42 (47.7%) | 24 (58.5%) |
| Moderate: score 2-3 | 37 (35.2%) | 5 (20.8%) | 35 (33.0%) | 7 (30.4%) | 32 (36.4%) | 10 (24.4%) |
| High: score >4 | 17 (16.2%) | 4 (16.7%) | 17 (16.0%) | 4 (17.4%) | 14 (15.9%) | 7 (17.1%) |
| Barthel-20, median (IQR)^#^ | 19 (17-20)* | 18 (17-19)* | 19 (18-20)* | 18 (17-20)* | 19 (18-20)* | 18 (17-20)* |
| AMPS motor, mean (SD)^1#^ | 0.97 (0.83)* | 1.38 (0.65)* | 1.07 (0.87) | 1.12 (0.48) | 1.12 (0.73) | 1.01 (0.91) |
| AMPS process, mean (SD)^1#^ | 0.75 (0.89)* | 1.35 (0.11)* | 0.84 (0.92) | 1.16 (0.38) | 0.83 (0.91) | 1.03 (0.72) |
| TUG score, mean (SD)^2#^ | 15.0 (11.2) | 15.9 (7.8) | 14.9 (11) | 16.5 (8.69) | 14.2 (10.3) | 17.4 (11.4) |
| 30s-CST, mean (SD)^3#^ | 6.9 (4.8) | 4.9 (4.9) | 6.7 (5) | 5.6 (3.7) | 7.0 (4.8) | 5.4 (4.9) |

^#^Barthel-20 n= 125, ^1#^AMPS n=83, ^2#^TUG n=110, ^3#^30s-CST n=116, *p<0.05

**Supplementary Table 4.** Differences between participants with a score of zero in domains and other participants

|  | D1. Cognition | | D3. Self-care | | D4. Getting along | | D5. Life-activities | |
| --- | --- | --- | --- | --- | --- | --- | --- | --- |
|  | No floor  n=102 | Floor  n=27 | No floor  n=82 | Floor  n=47 | No floor  n=82 | Floor  n=47 | No floor  n=102 | Floor  n=27 |
| Mean age, years (SD) | 80.8 (7.5) | 79.0 (8.8) | 81.6 (7.3)* | 78.4 (8.1)* | 80.6 (7.8) | 80.2 (7.7) | 81.2 (7.9)* | 77.5 (6.5)* |
| Female, n (%) | 56 (54.9%) | 12 (44.4%) | 48 (58.5%) | 20 (42.6%) | 45 (54.9%) | 23 (48.9%) | 59 (57.8%)* | 9 (33.3%)* |
| Comorbidity, n (%) |  |  |  |  |  |  |  |  |
| Low: score 0-1 | 54 (52.9%) | 12 (44,4%) | 42 (51.2%) | 24 (51.1%) | 43 (52.4%) | 23 (48.9%) | 52 (51%) | 14 (52%) |
| Moderate: score 2-3 | 30 (29.4%) | 12 (44.4%) | 26 (31.7%) | 16 (34%) | 27 (32.9%) | 15 (31.9%) | 31 (30.4%) | 11 (40.7%) |
| High: score >4 | 18 (17.7%) | 3 (11.1%) | 14 (17.1%) | 7 (15%) | 12 (14.6%) | 9 (19.2%) | 19 (18.6%) | 2 (7.4%) |
| Barthel-20, median (IQR)^#^ | 19 (17-20) | 20 (18-20) | 18 (17-20)* | 20 (19-20)* | 19 (17-20)* | 20 (18-29)* | 19 (17-20)* | 20 (19-20)* |
| AMPS motor, mean (SD)^1#^ | 0.99 (0.83)* | 1.41 (0.62)* | 0.81 (0.75)* | 1.42 (0.73)* | 0.91 (08.84)* | 1.31 (0.69)* | 0.86 (0.81)* | 1.66 (0.38)* |
| AMPS process, mean (SD)^1#^ | 0.88 (0.94) | 0.99 (0.43) | 0.89 (0.96) | 0.92 (0.68) | 0.80 (0.98) | 1.05 (0.84) | 0.78 (0.95)* | 1.23 (0.35)* |
| TUG score, mean (SD)^2#^ | 15.6 (10.4) | 13.3 (11.5) | 17.9 (12.0)* | 10.7 (5.7)* | 15.5 (9.3) | 14.5 (12.8) | 16.6 (10.4)* | 10.5 (10.3)* |
| 30s-CST, mean (SD)^3#^ | 6.0 (4.7)* | 8.6 (4.9)* | 4.9 (4.3)* | 9.4 (4.4)* | 5.8 (4.7)* | 7.7 (4.9)* | 5.5 (4.6)* | 10.3 (3.7)* |

^#^Barthel-20 n= 125, ^1#^AMPS n=83, ^2#^TUG n=110, ^3#^30s-CST n=116, *p<0.05
