# Supplementary figures and images for: Construct Validity and Clinical Utility of World Health Organization Disability Assessment Schedule 2.0 in Older Patients Discharged From Emergency Departments
Source: Front Rehabil Sci. 2021 Aug 17;2:710137. doi: 10.3389/fresc.2021.710137 (PMC9397984; doi:10.3389/fresc.2021.710137)

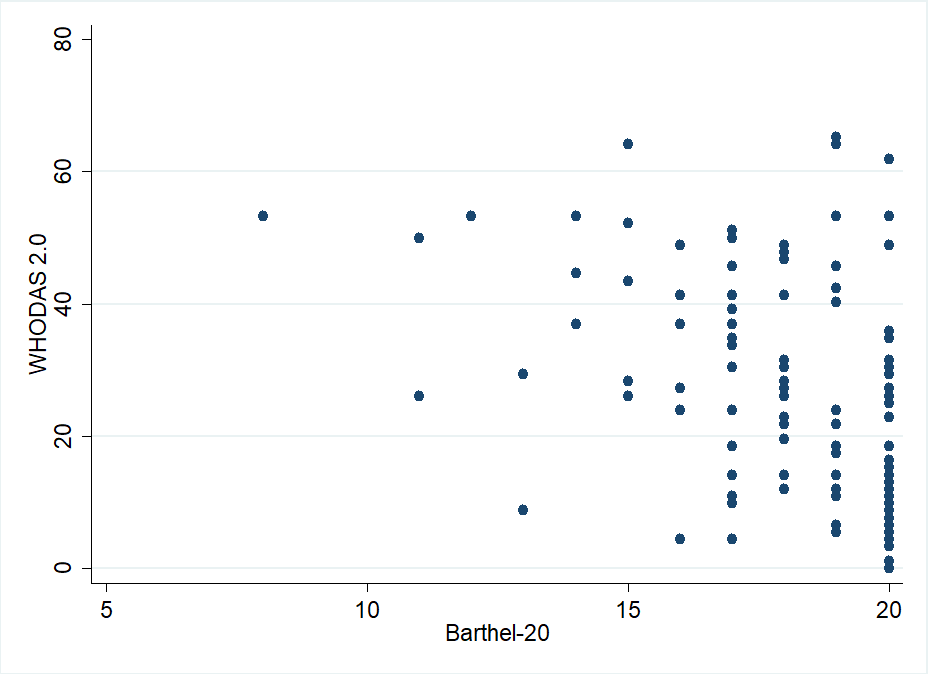

Supplement: Supplementary file 2 [file Image_1.TIF]

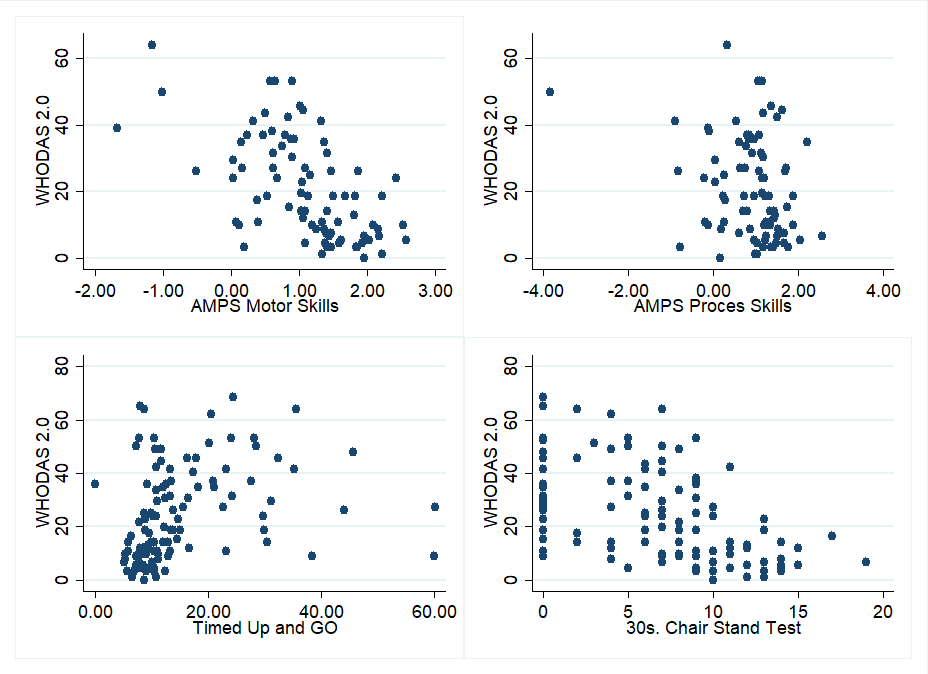

Supplement: Supplementary file 3 [file Image_2.TIF]
